# Neural correlates of positive and negative performance feedback in younger and older adults

**DOI:** 10.1186/s12993-015-0062-z

**Published:** 2015-04-16

**Authors:** Barbara Drueke, Lydia Weichert, Thomas Forkmann, Verena Mainz, Siegfried Gauggel, Maren Boecker

**Affiliations:** Department of Medical Psychology and Medical Sociology, University Hospital of RWTH Aachen University, Pauwelsstr. 19, 52074 Aachen, Germany

**Keywords:** Aging, Amygdala, fMRI, Performance feedback, Reward, Striatum

## Abstract

**Background:**

Recent studies with younger adults have shown that performance feedback can serve as a reward, and it elicits reward-related brain activations. This study investigated whether performance feedback is processed similarly in younger and older adults and whether there are differential aging effects for positive and negative performance feedback.

**Methods:**

We used event-related *f*MRI in a choice reaction-time task and provided performance feedback after each trial.

**Results:**

Although younger and older adults differed in task-related activation, they showed comparable reward-related activation. Positive performance feedback elicited the strongest striatal and amygdala activation, which was reflected behaviorally in slightly faster reaction times.

**Conclusions:**

These results suggest that performance feedback serves as a reward in both younger and older adults.

## Background

It has been shown that performance feedback can serve as an extrinsic (e.g., monetary) reward and that it engages corresponding brain regions [1,2]. The reward system has been described as a highly interconnected network of brain areas that include the striatum, amygdala, orbitofrontal and medial prefrontal cortex, and the dopaminergic mid-brain (for a review see [3]). Using a time-estimation task, Tsukamoto et al. [2] found that true performance feedback elicited stronger hemodynamic responses in the striatum, thalamus, and insular cortex than randomized feedback, which was not related to the participants’ time estimation performance in the task. They suggested that for humans performance feedback serves as an implicit reward. Aron et al. [1], Rademacher et al. [4], and Tricomi et al. [5] obtained similar results. Tricomi et al. [5] compared performance feedback processing in a learning task with reward processing in a guessing task and found similar activation in the caudate nucleus on the two tasks. Using smiling human faces in an incentive delay task, Rademacher et al. [4] compared monetary reward and performance feedback and found that anticipation of either monetary reward or performance feedback activated brain structures in the reward pathway, including the ventral striatum. Results indicate that feedback may serve as reward for participants but yet it is not clear whether feedback processing depends on aging processes, i.e. whether the elderly process feedback information in the same way as younger people. Cognitive domains like learning or memory normally change during aging and as these changes have implications for maintaining independence and quality of life, it is important to get knowledge of the normal changes in cognition that occur in aging to provide an essential background to understanding of interventions to optimize cognition in older adults. Psychological factors as for example memory training or feedback interventions were identified as important determinants of cognition in aging [6]. It has been demonstrated in memory training classes that elder like young adults can improve their performance on cognitive tasks [7]. Memory trainings for healthy older adults typically teach mnemonic strategies, concentration and attention, relaxation, self-monitoring, feedback, motivation, problem solving, and personal insight that have succeeded in improving memory performance [8].

Another important aspect is feedback valence that seems to modulate reward-related activity during feedback. Several studies have found higher striatal activation during positive feedback than during negative, whereas no areas were more strongly activated during negative feedback than during positive (e.g., [9-11]). On the other hand, Aron et al. [1] reported stronger midbrain activation during negative feedback than during positive, whereas positive feedback did not yield stronger activation than negative feedback. These heterogeneous results need further clarification regarding relevant brain areas for positive and negative feedback. Also, the above mentioned studies investigated only young adults, but it would also be important to examine brain processing during performance feedback among the older adults.

It has been shown that aging is associated with deterioration of brain functioning (e.g., [12,13]). In particular the dopamininergic system, which is associated with reward processing, is susceptible to aging (e.g., [14,15]). Additionally, Drueke et al. [16] found evidence that feedback positively influenced performance in an executive function task in younger but not necessarily in older adults. Performance feedback means to inform a person about how his behavior is perceived, realized and experienced by another person. Thereby, feedback includes information about the results, effects and consequences which may be useful if someone has to do appropriate adjustments of his own behavior. With regard to aging it is important to know if both younger and older individuals process feedback in the same way. Older adults often suffer from deficits in cognitive and motor skills because of neurological or physical diseases which might be treated with specific trainings. One useful intervention in such trainings is performance feedback which improves performance of daily activities and, as a consequence, influences quality of independent living.

We were interested in determining whether the brain reactions that have been observed during feedback among younger participants also occur in older adults in order to optimize possible intervention strategies to improve cognitive aging. Our aim was to use fMRI to compare younger and older participants’ neural processing during positive and negative feedback. We chose a choice reaction-time task with individual reaction time windows to insure equal distribution of real positive and negative performance feedback to participants about their reaction times. We also investigated the effects of performance feedback on subsequent reaction times and accuracy of performance in the choice reaction-time task. We hypothesized that as with younger adults, performance feedback given to older adults would elicit striatal activation indicating that performance feedback serves as a reward. Because several studies have shown weaker striatal activity in older adults during reward association learning (e.g., [17-19]), we expected weaker striatal activity in older than in younger adults during performance feedback. We also hypothesized that positive feedback would elicit stronger activation in the striatum compared to negative feedback.

## Methods

### Participants

Participants were recruited through a press release in a local newspaper and posters placed in strategic locations. Healthy younger male adults (N = 16) between the ages of 20 to 38 years (M = 25.2 ± 5.0) and healthy older male adults (N = 16) between the ages of 62 to 77 years (M = 69.4 ± 3.8) participated in the present study. Each participant completed a health questionnaire including questions about major life areas (e.g. physical and mental health and prescription medications, education) that served to identify participants who met the inclusion criteria. Individuals with neurological or mental disorders and those taking medications that affect their cognitive functioning (e.g. anticholinergic drugs, beta blocker) were excluded. Also excluded were participants who did not fulfill the inclusion criteria for investigation with functional magnetic resonance imaging (fMRI, e.g. anyone with any metal in the body as cardiac pacemakers, aneurysm clips, cochlear/retinal implants, hearing aids, tattoos, metal plats/pins/screws on bones). All participants were right-handed and were informed about the objectives and procedure of the study. The study protocol was approved by the local ethics committee and all participants gave written consent. They were paid a small allowance.

### Experimental paradigm

A computer-based choice reaction time task was employed, which was a modified version of the flanker task (e.g., [20]). Participants were required to indicate whether an arrow presented in the center of a computer screen pointed to the left or to the right by pressing the corresponding key on the keyboard with their right hand. The target arrow was flanked on either side by two arrows pointing in the same or the opposite direction. Participants were instructed to respond as quickly and accurately as possible. The task was performed while participants were in an MRI scanner. Before the task began, participants were given a practice block of 10 trials. They could repeat the practice trials until they felt that they were familiar with the task. To assess participants’ baseline performance, they performed an offline baseline block comprising 48 trials. Thereafter, in the scanner participants performed six experimental blocks comprising 48 trials each.

**Figure 1:**
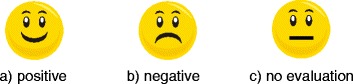
Three feedback conditions.

Participants were given feedback after each individual trial to assess its effect on their performance [21]. On 67% of the trials, participants were given feedback, which was either positive or negative depending on their performance. In the remaining 33% of the trials, participants’ performance was not evaluated (neutral condition). Feedback was provided by presenting silhouette faces with a positive or a negative valence. The three feedback conditions are depicted in Figure 1.

**Figure 2:**
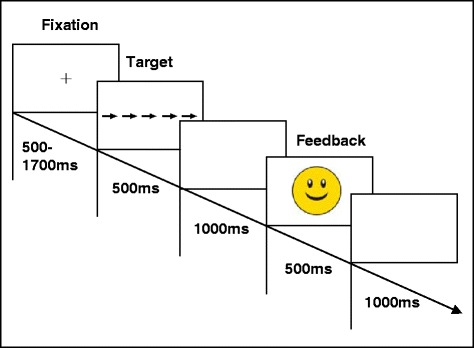
Trial flow in the paradigm.

Each target stimulus was placed in the center of the screen, at a visual angle of 2.86° horizontally and 0.24° vertically. On each trial, a fixation cross was first presented for a variable interval that lasted from 500 to 1700 ms. Then, the target was shown for 500 ms and was followed by a blank screen for 1000 ms. Subsequently, feedback was displayed for 500 ms followed again by a blank screen for 1000 ms. The next trial started with the presentation of the fixation cross. The sequence of trial events is depicted in Figure 2.

Performance feedback was evaluated relative to each participant’s reaction times and was continually adjusted throughout each participant’s performance. Individual reaction time terciles were computed across the last 48 trials and were updated as each new trial was performed. This dynamic tracking was implemented to take into account variations during the trials, for example, those resulting from practice or fatigue, and to insure an equal distribution of positive and negative feedback throughout the trials.

If the participant’s response was correct and corresponded to the lower reaction time tercile, a smiling face was presented to indicate good performance. If the participant’s response was incorrect or corresponded to the upper reaction time tercile, a frowning face was presented, which indicated relatively poor performance. On the trials that were not evaluated, a face with a neutral expression was presented. The middle tercile was used to balance the quantities of positive and negative feedback, i.e. the participant received positive feedback if only negative feedback had previously been given, and vice versa.

Participants were instructed to react as quickly as possible and to collect as many smiling faces as possible. They were told that if their response was correct and the reaction time matched their own better level of performance, they would receive positive feedback. If their response was incorrect or their reaction time matched their own poorer level of performance, they would receive negative feedback. They were also told that randomly in one-third of the trials, their performance would not be evaluated indicated and a neutral face would be presented. After performing the task in the scanner, participants completed a questionnaire on which they were asked how much the feedback corresponded to their own evaluation of their performance.

### Procedure

Participants first completed a screening questionnaire about the status of their health. No participant had to be excluded for health reasons, and no participant had a history of a neurological or a psychological disorder. While participants were seated approximately 60 cm in front of a computer screen, the practice and baseline blocks of trials were first conducted offline. In the MRI scanner, the six experimental blocks were then performed, and after each block, there was a rest period of approximately 15 sec. A software package (Neurobehavioral Systems, San Francisco, CA) was used to present the stimuli. Participants were instructed to respond as quickly and accurately as possible. The six experimental blocks lasted about 25 min.

### Image acquisition

The MRI data were acquired using a 3 Tesla Siemens Trio (Siemens Medical Solutions, Erlangen, Germany) equipped with a standard head coil. Changes in blood oxygenation level-dependent (BOLD) T2*-weighed MR signals were measured using a gradient echo-planar imaging (EPI) sequence (42 slices, 2.5 x 2.5 x 2.5 mm voxels, 10% gap, TR = 2.4 s, TE = 30 ms, flip angle = 90°, 64x64 matrix, FOV 220 mm, interleaved acquisition).

### Statistical analysis

For analysis of the behavioral data, only correct trials with a reaction time between 200 and 2000 ms were included. In addition, an outlier analysis was performed. Trials with reaction times 2.5 standard deviations above or below the mean were not included. A repeated-measures ANOVA with factors *age group*, and *feedback valence* was conducted, and Greenhouse-Geisser *F*-values are reported. Reaction times on correct trials just after the positive or negative feedback were analyzed. When effects were significant, post hoc Tukey HSD tests were computed, and corrected *p*-values are given, as are effect sizes.

The imaging data were analyzed using the SPM5 software package (Wellcome Department of Cognitive Neurology, London). For each participant, all functional images were spatially realigned to the first volume to correct for interscan head movements, interpolated in time to correct for differences in slice acquisition time, normalized to a standard MNI template, and smoothed with a Gaussian kernel of 8 mm full-width half maximum to accommodate intersubject anatomical variability.

At the first level, data were analyzed by modeling six experimental conditions (2 x 3 conditions) using the canonical hemodynamic function (hrf) in SPM5, time-locked with the presentation of feedback. Only correct trials with a reaction time between 200 and 2000 ms were included. A full factorial model was computed at the second level with age group (younger/older adults) and feedback valence (positive/negative/neutral) as the two factors. A *p*-value of 0.05 was set for all analyses after correcting for family-wise error (FWE) across the whole brain and setting a minimal cluster size of 5 contiguous voxels. If activation was significant, directional *T*-tests were conducted. All contrasts were masked inclusively with the minuend (*p* < 0.05 uncorrected). Finally, coordinates of activations were transformed from MNI to Talairach space [22].

Furthermore, ROI analyses were performed on the amygdala, the caudate and the nucleus accumbens since previous literature (e.g., [9]) has suggested a greater involvement of these structures in processing of positive compared to negative feedback. For the caudate and the amygdala, 2^nd^ level contrasts were calculated within these regions as defined by the automated anatomical labeling (AAL) [23]. Anatomical labeling provided in the tables was performed with help of the AAL-coordinates provided by the WFU-Pickatlas [23]. For the ROI of the nucleus accumbens, an 8 mm sphere was centered at Talairach coordinates [±10, 8, −5].

## Results

### Behavioral data

#### Feedback ratio and ratings

Neutral uninformative feedback was pseudorandomly given on 96 trials (33.3%). On average, positive feedback was given on 94.3 trials (*SD* = 3.5; 32.8%), and negative feedback on 97.7 trials (*SD* = 3.5; 33.9%). Analysis of variance for repeated measures with the factors feedback and age revealed a significant main effect of feedback (F = 7.35, p < .01, η^2^ = .197). Post-hoc tests showed that the number of trials on which positive, negative and neutral feedback were given differed significantly (p < .05). Post-hoc tests were corrected for multiple comparisons (Bonferroni). There was no difference between younger and older adults regarding positive and negative feedback ratios.

#### Reaction time and accuracy

Descriptive statistics of reaction times and accuracy, separated for younger and older adults, are presented in Table 1. An ANOVA with reaction times as dependent variable yielded no significant age group by feedback interaction (F2,60 = 1.84, p > .05; η^2^ = .058). Significant main effects of both age group (F1,30 = 47.83, p < .001; η^2^ = .615) and feedback valence (F2,60 = 4.27, p < .05; η^2^ = .125) on subsequent reaction times were found. Older participants displayed slower responses (M = 501 ms ± 36) than younger participants (M = 384 ms ± 58). Regarding feedback, post-hoc Tukey HSD tests showed that after positive feedback (M = 440 ms ± 77), reaction times were faster than after receiving neutral feedback (M = 446 ms ± 80; p < .05). The difference between positive and negative feedback (M = 445 ms ± 76) was not significant (p = .07). When regarding the effect sizes of feedback valence separated by age group, no effects could be found in younger adults for positive as compared to neutral (ES = 0.05) and to negative (ES = 0.10) feedback. In older adults, small effect sizes can be reported for positive versus neutral (ES = 0.23) and versus negative (ES = 0.10) feedback.

**Table 1:** **Arithmetic means and standard deviations of reaction times (RT) and accuracy of performance in younger (** ***N*** **= 16) and older (** ***N*** **= 16) adults**

	**Younger**	**Older**	**All**
Mean RT (ms)			
Neutral Feedback	384 (61)	508 (38)	446 (80)
Positive Feedback	381 (59)	499 (35)	440 (77)
Negative Feedback	387 (57)	503 (38)	445 (76)
Accuracy (%)			
Neutral Feedback	91.5 (5.5)	93.4 (4.2)	92.5 (4.9)
Positive Feedback	91.1 (5.6)	90.9 (8.7)	91.0 (7.2)
Negative Feedback	92.2 (7.0)	91.1 (7.1)	91.7 (7.0)

Results of the repeated measures ANOVA with accuracy as dependent variable yielded neither a significant interaction (F2,60 = 1.84, p > .05; η^2^ = .058), nor main effects for feedback valence (F2,60 = 1,53, p > .05; η^2^ = .049) or age group (F1,30 < 1; η^2^ = .000) on subsequent accuracy.

### Imaging data

#### Interaction age group by feedback

A feedback by age group interaction revealed no significant activation for the set *p*-level of 0.05 after correcting for multiple comparisons across the whole brain.

#### Main effect of age group

The main effect of age group (*p* < 0.05, corrected for multiple comparisons) revealed bilateral activations in the precuneus and superior parietal lobule, the right middle frontal gyrus, right middle temporal gyrus and left lingual gyrus. Because significant activations were demonstrated for age group as a main effect, post-hoc directional t-tests were computed with older adults versus younger adults.

#### Older adults > younger adults

**Figure 3:**
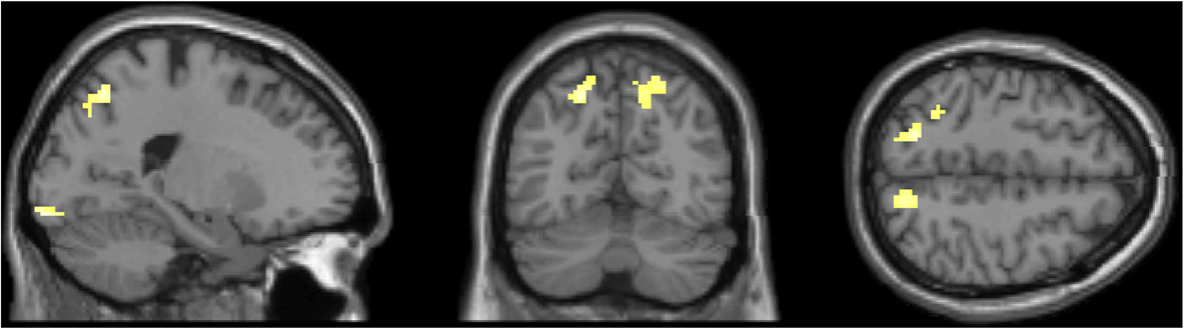
Older adults > younger adults (masked incl.), p = .05 (FWE-corrected), x = −20.0, y = 65.0, z = 50.0.

The results of the directional *t*-test older adults > younger adults (masked incl.) are depicted in Table 2. Older adults exhibited stronger activations bilaterally in the precuneus including the right superior and inferior parietal lobule as local maxima, the right middle frontal gyrus, right fusiform gyrus, right postcentral gyrus as well as in the left lingual gyrus. The activation clusters of local maxima are depicted in Figure 3. No suprathreshold voxels remained in the contrast younger adults > older adults. According to our hypothesis, we conducted a region of interest (ROI) analysis for the contrast young > old which yielded no significant activation clusters in striatal areas.

**Table 2:** **Activation contrasts in older vs. younger adults**

**Area**	**BA**	**x**	**y**	**z**	**voxels**	**t-value**
OLDER > YOUNGER						
Lingual gyrus (L)	17	−17	−95	−6	55	6.08
Middle frontal gyrus (R)	6	35	−2	62	23	5.95
Precuneus (L)	7	−21	−61	49	59	5.59
Precuneus (R)	7	15	−66	47	134	5.45
Superior parietal lobule (R)	7	25	−58	56	-	5.28
Inferior parietal lobule (R)	40	32	−51	56	-	5.20
Superior parietal lobule (L)	7	−32	−46	46	32	5.38
Fusiform gyrus (R)	37	42	−57	−14	6	5.15
Postcentral gyrus (R)	40	40	−34	57	7	5.03
YOUNGER > OLDER						
No suprathreshold clusters

#### Main effect of feedback

The main effect of feedback (p < 0.05, corrected for multiple comparisons) revealed bilateral activations in the putamen, medial frontal gyrus, precentral gyrus and lingual gyrus as well as in the left thalamus, anterior cingulate (ACC), superior frontal gyrus, the right precentral and superior temporal gyrus.

Because significant activations were demonstrated for feedback as a main effect, post-hoc directional T-tests with pairwise comparisons of positive, negative and neutral feedback were computed. Data from younger and older adults were combined.

#### Positive feedback > negative feedback

**Figure 4:**
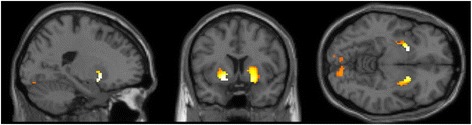
Positive feedback > negative feedback (masked incl.), p = .05 (FWE-corrected), x = −20.0, y = 7.5, z = −7.5.

Positive feedback elicited stronger activations as compared to negative feedback bilaterally in the putamen and in the left amygdala, the lingual gyrus, in the right medial and left superior frontal gyrus and in the thalamus (see Table 3). The activation clusters *of local maxima* are illustrated in Figure 4. No suprathreshold activation clusters remained for the opposite contrast (negative feedback > positive feedback).

**Table 3:** **Activation contrasts between positive vs. negative and neutral feedback**

**Area**	**BA**	**x**	**y**	**z**	**voxels**	**t-value**
POSITIVE > NEGATIVE						
Putamen (L)	49	−20	7	−7	101	12.39
Amygdala (L)		−15	4	−11	-	11.99
Putamen (R)		20	9	−7	253	10.52
Lingual (L)	18	−15	−85	−6	109	6.40
Lingual (R)	17	12	−85	0	-	6.01
Medial frontal gyrus (R)	6	5	−2	60	16	6.25
Superior frontal gyrus (L)	8	−17	37	51	19	5.77
Thalamus		0	−5	5	10	5.53
NEGATIVE > POSITIVE						
No suprathreshold clusters						
POSITIVE > NEUTRAL						
Putamen (L)	49	−17	7	−7	20	6.32
Amygdala (L)		−15	4	11	-	6.01
Putamen (R)	49	20	9	−7	14	5.71
NEUTRAL > POSITIVE						
No suprathreshold clusters						
NEUTRAL > NEGATIVE						
Putamen (R)	49	25	0	5	13	5.24
Fusiform gyrus (L)		−27	66	−11	11	5.26
Lingual gyrus (L)	18	−12	−85	−6	8	5.00
NEGATIVE > NEUTRAL						
No suprathreshold clusters

Furthermore, ROI analyses were performed on the amygdala, the caudate and the nucleus accumbens. The comparison between positive and negative feedback (pos > neg) ROI analyses revealed significant bilateral activations for the amygdala (x = 22, y = 4, z = −13; x = −25, y = −1, z = −10), the right caudate head (x = 15, y = 12, z = 2), and bilateral nucleus accumbens (x = 12, y = 10, z = −10; x = −18, y = 8, z = −8).

#### Positive feedback > neutral feedback

**Figure 5:**
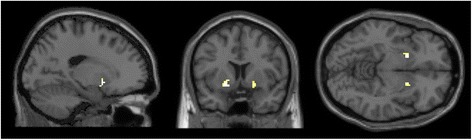
Positive feedback > neutral feedback (masked incl.), p = .05 (FWE-corrected), x = −17.5, y = 7.5, z = −7.5.

When contrasting positive and neutral feedback, it was demonstrated that positive feedback yielded stronger activations bilaterally in the putamen including the left amygdala as a local maximum (see Table 3), the activation clusters are depicted in Figure 5. No stronger activations were found for neutral feedback.

#### Neutral feedback > negative feedback

**Figure 6:**
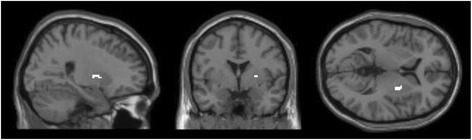
Neutral feedback > negative feedback (masked incl.), p = .05 (FWE-corrected), x = 25.0, y = 0.0, z = 5.0.

The comparison of neutral feedback with negative feedback showed stronger activations for neutral feedback in the right putamen, the left fusiform and lingual gyrus. Figure 6 illustrates significant activations. The opposite contrast (negative feedback > neutral feedback) yielded no suprathreshold activation.

## Discussion

The present study compared older and younger adults on positive and negative performance feedback processing using fMRI. The imaging results did not show an age-by-feedback interaction, which indicates that younger and older adults process positive and negative feedback similarly. Although older adults displayed stronger activation in several brain areas than younger adults, these areas were neither task-related nor associated with the reward system. Older adults displayed stronger occipital and parietal activation involved in visual and spatial processing. Additionally, they showed stronger frontal lateral premotor (BA 6) activation, which has been associated with the selection of movements (e.g., [24]).

It has been suggested that increased activation, especially increased bilaterality, would help older adults to counteract age-related neurocognitive decline. This account is also known as the compensation hypothesis [25,26]. A more differentiated view proposes that compensatory activity may be effective only if it can play a complementary role in task performance [27]. Because older adults in the present study showed stronger activation in brain areas associated with both movement selection and visual and spatial processing of stimuli, it seems reasonable to conclude that the recruitment of these brain areas had a compensatory function and helped the older adults in performing the task.

The behavioral data are consistent with the imaging results in that they also show an effect for aging. The older adults responded much more slowly than the younger adults. This age-related slowing has been demonstrated in a variety of studies and has been interpreted in terms of a slower information processing system in older adults (e.g., [28-31]). It can be concluded that because of their slower information processing system, the flanker task was more demanding for the older adults. This might have resulted in a compensational recruitment of brain areas that enhanced visuospatial processing of stimuli and motor planning.

Contrary to our hypothesis, older adults did not show weaker striatal activity than younger adults. This result suggests that in older adults, reward-related performance feedback processing is intact and comparable to that of younger adults. The behavioral results support this interpretation in that both younger and older adults had marginally faster reaction times after positive feedback than after negative or neutral feedback. It can be inferred that positive feedback about task performance served as an extrinsic reward and led to greater effort (see [32]) and faster reaction times.

The effect sizes were small perhaps because the study was optimized for the fMRI rather than the behavioral measures. Dynamic tracking in the task was employed to help insure that there was an equal distribution of positive and negative performance feedback over the course of the experiment. Thus, participants who improved during the task still received an equal proportion of positive and negative feedback. At the beginning of the task, participants might have received positive feedback for slower reaction times, but faster reaction time might have eventually led to negative feedback due to the continuous updating of reaction time terciles. This effect of dynamic tracking might have attenuated the impact of the feedback on a behavioral level. Nevertheless, there were marginally faster reaction times for positive performance feedback, indicating that it did serve as a reward.

These findings are also reflected in the imaging results, which revealed that positive feedback elicited higher activation than negative or neutral feedback in the putamen and the amygdala. Thus, the results confirm that both the dorsal striatum and the amygdala are involved in the neural processing of performance feedback in both younger and older adults.

It has previously been shown that the dorsal striatum is involved in reward delivery (e.g., [33,34]). On the other hand, it has been suggested that the dorsal striatum responds to the reinforcement of an action that is contingent on behavior rather than to reward delivery itself [35]. In the present study, both the behavioral and the imaging results support our hypothesis that performance feedback serves as a reward and elicits striatal activation. The amygdala is associated with the processing of the emotional valence of stimuli [36,37] and is known to have connections to the striatum [38]. The stronger amygdala activation in processing positive feedback might, therefore, be due to a coding for emotional valence. This stronger amygdala activation after positive feedback is not in line with previous research mostly reporting a greater magnitude of activation for negative than for positive emotional stimuli methods [39,40]. Other researchers hypothesized that amygdala activation might code emotional intensity rather than, or in addition to, emotional valence [41,42]. This hypothesis is also supported from results of lesion studies: Berntson et al. [43] found the amygdala to be important for the registering of the arousal or emotional impact in patients with amygdala lesions. It is possible that the amygdala activation in our study might be explained by the participants’ general arousal during the task. We also found greater activation in medial frontal gyrus after positive feedback which might be related to amygdala activation as well: Banks et al. [44] found a task-dependent functional connectivity between specific areas of the frontal cortex and the amygdala. Perlman et al. [45] found greater amygdala activation in patients with major depression than in control patients and less connectivity between amygdala and medial prefrontal cortex.

The present results are consistent with a recent study [9] which also found that positive feedback elicited higher activation in both the putamen and the amygdala than negative or uninformative feedback. Somewhat unexpectedly, the neutral feedback condition elicited higher putamen activation than the negative feedback condition, although the neutral trials were not supposed to be rewarding. This might be explained by the fact that feedback condition that was intended to be neutral was not always perceived as neutral. Participants reported that they were happy to receive “neutral” feedback after a subjectively slow response and disappointed after a subjectively fast response. In fact, therefore, the “neutral” feedback was a mixture of positive and negative feedback, and it elicited stronger reward-related activation than negative feedback. It should be noted that the neutral feedback was not contingent on participants’ behavior, but it was given completely randomly. Thus, the stronger dorsal striatal activity seems not to be associated with the reinforcement of behavior, but rather with reward delivery itself. This contradicts the suggestion that the dorsal striatum responds to behavior-reward contingency [35]. Against our hypothesis we found no stronger striatal activation for younger than for older adults.

In addition to reward-related activation, positive feedback elicited stronger activation than negative feedback in the visual cortex—indicating enhanced visual processing of stimuli—and in areas associated with the planning of movements (BA 6/ 8). Additionally, stronger activation in the thalamus was found, which might be related to the integration of feedback processing and preparation faster reactions after positive feedback. This additional task-related activation corresponds to the behavioral results, which showed that after positive feedback, reaction times improved in both younger and older adults.

Finally, it should be acknowledged that the present study had certain limitations. For example, we tested relatively young along the older adult life span continuum who are more likely to engage in cognitively challenging activities which have beneficial effects on cognitive functioning [30]. Furthermore, we chose to investigate only male participants as some researchers found evidence for changes in cognitive functioning that might be due to hormonal state in women. For example see Weis et al. [46] who found changes in results of behavioral data as well as in imaging data due to womens’ menstrual cycle phase. The results of this study therefore, cannot be generalized to older women. Another important aspect refers to the connectivity between specific brain areas (e. g. amygdala and frontal cortex) which may vary between younger and older adults. Future studies should address this issue because the connectivity may be less or greater in older adults.

Taken together, the results indicate that performance feedback can serve as a reward in both younger and older adults. Despite having a slower information processing system, older adults were still able to improve their performance after receiving positive feedback. Additionally, the imaging results supported the roles of the striatum and the amygdala in performance feedback processing. Inasmuch as no difference in reward-related processing of performance feedback was found between younger and older adults, it can be inferred that younger and older adults process performance feedback similarly. Activations found in the dorsal striatum seem to be associated with the processing of reward delivery rather than behavior-reward contingency. Stronger neural activation in older than younger adults seems to reflect task-specific demands and points to compensatory recruitment of areas associated with visual and premotor processing.

## Conclusions

The present study indicates that the behavioral and neural processing of positive and negative performance feedback is preserved in older adults. It was shown that positive performance feedback can serve as a reward in both older and younger adults. These results have important clinical implications for intervention studies aimed at improving cognitive performance in older adults. Whereas an extrinsic reward such as money would be unsuitable to use in cognitive training, performance feedback can easily be implemented in a training procedure. It has the additional benefit of being able to tap a neural mechanism that is in tact in older adults.
